# Progressive and Retrograde Metamorphosis of Bones and Teeth

**Published:** 1874-10

**Authors:** S. P. Cutler


### DENTAL REGISTER.

VOL. XXVIII.] OCTOBER, 1874. [No. 10.

### PROGRESSIVE AND RETROGRADE METAMORPHOSIS OF BONES AND TEETH.

BY S. P. CUTLER, M. D., D. D. S.

*Read before the District Dental Society, of the Seventh Judicial District, of the State of New York. Rochester, June 2d and 3d,* 1874.

Growth and development of bones and teeth are very well understood by physiologists in general.

Bones and teeth, like all other tissues, are amenable to laws of development and growth up to a certain period of maturity, when further advances cease, and a stage of longer or shorter duration, differing somewhat in different individuals, remains stationary; after which time, a retrograde movement advances, never halting until life and vitality of the osseous tissues are crowded out by displacement, so to speak, and drag down the vitality of the soft tissues with them.

In the fœtus at a certain stage of development, there are no bones yet formed or ossified, and, at other certain stages, after the matrix or bone cartilage has been laid down or formed, points of ossification begin to take place.

The primary bone cartilage becomes differentiated into true bone, by a process of saturation of the cartilage with triple phosphate, or neutral phosphate of lime, or calcic phosphate, viz:

| | |
|---|---|
| $3Ca\ (PO_4)$ New notation<br>$3CaO,\ PO_5$ Old notation | Also a small per cent of calcic carbonate $CaO,\ CO_2$ |

This process of differentiation or change of cartilage into bone may be regarded in the first instance as osmotic absorption of the salts of lime, governed by some molecular law, not yet quite clearly understood of some vital or other affinity existing between the animal and inorganic elements, resulting in a molecular hardening of the tissues, that may be likened to the process of putrefaction of wood and animal structures, outside of living bodies.

The cartilage being devoid of nerves and blood vessels, has, in consequence, very little vital existence, allowing the encroachment of lime or earthy salts to enter, and there remain and harden into true bone; the earthy salts become insoluble, the same as crystals of these salts outside of the body.

Unlike true petrifactions, bones are subject to growth and change,

In the fœtus, after the cartilages of the long bones have become ossified, if no further structural change took place, the bones might still grow by added layers on their surfaces.

But the hollows of the long cylindrical bones, filled with marrow, would remain the same size at maturity they had in the fœtus, which is not the case; as these canals increase in size as the bones grow, outwardly, in certain definite proportions.

This seems to be a double process or metamorphosis, as we find the marrow in the adult bones quite as large as the entire fœtal bone at one period. It is this process of change we know so little about; of the law or process of removal we know comparatively nothing, though evidently a vital process. This process of waste or enlarging of canals in the cylindrical bones might be explained in this way for want of better knowledge on the subject. We know that the fœtal bones are less dense or hard than adult bones, and the earthy salts proportionately less to the animal proportions, the union also less perfect and resistant.

In consequence, the earthy salts are more easily removed by any agency capable of bringing about such removal. It is hardly probable or possible for the process of absorption to take away the salts, even at this early age, much less so as the child advances in years. In accordance with ideas long since formed, I will suggest a few thoughts as to the cause of this molecular change.

We may suppose the marrow in the bones to contain just enough acid of some kind to take away an equivalent of lime, and leave an acid soluble salt of lime remaining which would be readily taken away by absorption and removed by the kidneys from the body; as it is not certain that this lime would not again be converted in the organism into the triple phosphate, though such may be the case, as many might argue.

This acid, or bi-phosphate may be given thus:

$2CaH,(PO_4)_2$ $3H_2O$ New notation

$2CaO,HO,PO_5$ $3HO$ Old notation.

In this way, we may imagine, molecule by molecule, removed at the surface of the marrow, and the canal enlarging; at the same time growth is taking place at the surface of the bones, keeping pace, *pari passu*, with the growth of the marrow until maturity. To fully comprehend the structural difference between bone and cartilage, they should be examined under the microscope, as they have scarcely any resemblance.

As the bone cartilage ossifies, hardens and becomes heavier, the cartilage itself does not enlarge, but remains the same in outline. As growth takes place, new layers of cartilage are added and saturated with lime more or less completely varying somewhat at different ages.

There is room thus in the structure of the cartillage to take, in by osmotic force lime salts, like water taking up a certain amount of sugar without enlarging the bulk or volume, which may be explained under the head of solutions.

The removal of one equivalent of the triple phosphate may be explained thus: Any acid coming in contact with bone, whose adhesive affinities are greater for lime than the entire cohesive attraction of the liquid molecules for each other, complete solution of the solid or a certain equivalent of it takes place. But if the adhesive force between the liquid and solid be greater than one-half of the cohesive force of the liquid molecules for each other, then wetting only takes place, as when a piece of gold is put into mercury, or a piece of dry wood into water.

When the adhesive attraction between any solid and liquid is less than half the entire cohesive attraction of the liquid molecules for themselves, no wetting takes place; as when glass and mercury come in contact, tallow and water.

The same explanation, it appears to me, should equally apply to the cohesive attraction of molecules for each other, both in solids and liquids, and not to liquids alone as is generally understood to be the case.

In simple solutions proper, there are no chemical changes, or change of properties of either the liquid or solid, the solid becomes liquid, and the liquid becomes more dense in having a greater specific gravity than before.

When any salt is formed as the union of an alkali and an acid or acid and oxide a total change of properties of both takes place by mutual saturation, and something new is formed differing from either compound.

What I wish to arrive at is this, when a tooth decays, acidity of the mouth is the disturbing factor, which I shall attempt to explain on the principle already given, viz: by a process similar to the formation of a solution, the difference being a chemical change in the one and not in the other. When any acid in the mouth, having a greater affinity for an equivalent of lime of the tooth substance than the molecules or atoms of such an acid has for one another, then a union of acid and lime of the tooth takes place, forming a neutral salt of lime, as nitrate, sulphate, acetate or hydrochlorate of lime, as the case may be, owing to the nature of the oral acid.

This, to me, seems to be a satisfactory explanation of dental caries; the same explanation equally applies to all other chemical changes of whatever character.

The chief difference between the formation of a simple solution and a salt is the difference in the adhesive attraction in comparison to that of cohesive attraction as explained in this paper. This applies to common salts not the halogens, in the latter case, only two simple elements are employed. Simple solutions may be broken up by evaporation alone, as a rule, not so in the case of a salt.

When one equivalent of lime is recovered from the triple phosphate of the tooth, it leaves behind a bi-phosphate of lime which is soluble and easily washed away by the saliva, and the cartilage gives away, in some cases, no doubt, by oxidation.

In some forms of decay the cartilage remains intact, and the lime only removed, which serves to destroy the parts so affected. In some instances, sensibility remains in the animal portions after removal of lime, and some visionary minds may imagine a second differentiation of this cartilage by ossification or dentification under favorable circumstances by proper management and treatment, though I must confess I am not that sanguine in the matter. Still that would be a most desirable result, and would come under the head of reproduction or restoration of lost part, a forward after a retrograde metamorphosis. Such a condition of things sometimes takes place in the bones.

Some have advanced the opinion that parasites play a mischievous part in dental caries, such as the leptothrix buccalis, denticolæ and oidium albicans.

The oidium, so far as my knowledge goes, are the most numerous. The protococens dentalis and other parasites are to be seen at times. The denticolæ mentioned above, I do not regard as parasites at all or even as organisms.

Some have advanced the idea, that alkalies act on the teeth, and cause caries. These are thought to act first on the cartilaginous portions, then the lime breaks down. They certainly could not claim that alkalies could in any way act on the lime, unless it be by combining with the phosphoric acid, by virtue of a stronger affinity than that of the lime of the tooth. An alkali of sufficient strength to act in this way would necessarily corrode the lining membrane of the mouth.

Let any dentist put a tooth into almost any diluted acid, and another into any diluted alkali, and let remain sometime. He will find more chemical action between the acid and tooth than between the alkali and tooth.

In dental caries, we more often find an acid reaction in the mouth than the opposite condition or even neutral.

Molecular polarity, no doubt, governs all molecular change; and all existing molecular or atomic combinations must have their polarities reversed or there can be no change, no molecular motion, no recurring event. All chemical action or change means molecular disturbance and breaking up of preexisting affinities, and the establishing of new and stronger ones.

In the living organism, all the retrograde movements, at least, are the result of purely chemical metamorphoses.

Returning to our osseous subject and the teeth, more especially, we find in tooth development a somewhat difficult set of phenomena, more than those of true bone.

When the basement membrane, between the enamel and dentine, is first laid down, the double process of development of both outward and inward begins. This starting point, being permanent, only extends itself as the crown is developed, the direction of dental growth being inwards and fangwards, never enlarging transversely. The enamel and cement only grow outwardly.

There is a stage of crown development and a stage of root development, somewhat independent of each other. When the eruptive stage commences, the crown is nearly, if not quite, complete. The enamel proper has no nerves except just at the dentinal surface, at which surface all the tubuli, including all branching from the pulp chamber outwards with their live true nerve fibrils, terminate. In some cases, at certain points, these fibrils are projected some distance into the enamel.

In all such cases, so far as my observation goes, there is undue sensibility at these points, on separating or filing down to such points, or excavating over the same.

In cases of tooth edge, as some writers term it, it would seem that the whole of the enamel becomes endued with true nerve sensibility, as in case of eating sour fruit, the acid in some way fires up the tooth, more especially if the enamel be worn a little. An alkali taken into the mouth removes the above sensation at once.

